# Holmium YAG laser ablation of a hemangioma involving a lower pole renal calyx - using the new-generation flexible ureteroscope URF type P5^®^: a case report

**DOI:** 10.4076/1757-1626-2-7780

**Published:** 2009-06-17

**Authors:** Kogenta Nakamura, Yoshiaki Yamada, Charles J Rosser, Shigeyuki Aoki, Tomohiro Taki, Nobuaki Honda

**Affiliations:** 1 Department of Urology, Aichi Medical University School of MedicineNagakute-cho, Aichi 480-1195Japan; 2 Department of Urology, University of FloridaGainesville, FLUSA

## Abstract

Hemangioma of the renal calyx is a rare disease, which is difficult to diagnose and an even greater challenge to treat. We report the use of the new-generation flexible ureteroscope, in the management of a 37-year-old Asian male with a lower pole renal calyx hemangioma, which was previously inaccessible.

## Introduction

Renal hemangioma involving the collecting system is a rare disease [1] and is difficult to diagnose and treat. Previous reports discussed the need for a more radical approach (total or partial nephrectomy) to treat these hemangiomas. The need for less invasive surgery should be considered in this cohort. However, there have been significant advances in the technology related to ureteroscopy over the past decade, limited data are available for the use of endoscopic treatment of hemangiomas involving the collecting system. We report a case of a previously inaccessible lower pole calyx hemangioma that we gained access with the new-generation flexible ureteroscope, URF type P5® (Olympus^TM^ Corp., Tokyo, Japan), and effectively ablated the hemangioma with the Holmium YAG (HoYAG) laser.

## Case presentation

**Figure 1.**
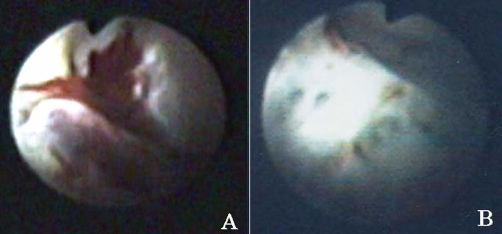
Ureteroscopy showed a hemangioma **(A)**. Findings after HoYAG laser ablation **(B)**.

A 37-year-old Asian male presented to his local urologist with a primary complaint of painless, gross, intermittent hematuria of two years duration. The previous urologist had performed and attempted ureteroscopy but was unable to fully examine the right collecting system. On presentation to our institute, the patient had abdominal ultrasonography, CT scanning and MRI, which demonstrated no abnormality. His urinary cytology was negative. Bloody efflux was observed from the right ureteral orifice on cystoscopy. The rest of the bladder appeared normal. Retrograde pyelogram of the right collecting system was within normal limits. The patient underwent right flexible ureteroscopy using flexible ureteroscope URF-P5. Dilation of the ureteral orifice was not necessary. The use of the URF type P5® ureteroscope allowed maximum mobility within the collecting system. This combined with fluoroscopic examination of the right collecting system confirmed complete pyeloscopy. As a result, a hemangioma of the right lower pole renal calyx was identified (Figure 1A). No papillary masses or another other lesions or stones were identified in the collecting system. Subsequently, we employed the HoYAG laser (laser probe Lumenis 200µm and a laser generator Versa Plus 80 W, Lumenis Inc., CA) to completely ablate this lesion in a rather bloodless field (laser settings-0.5 J, 5 Hz, total: 0.52 kJ). At the conclusion of the case, a double J ureteral stent was placed. Two weeks later, the ureteral stent was removed. Here 15 months after the procedure, the patient is asymptomatic. Furthermore, microscopic urinalysis and urinary cytology are negative for blood or malignancy, respectively.

## Discussion

**Figure 2.**
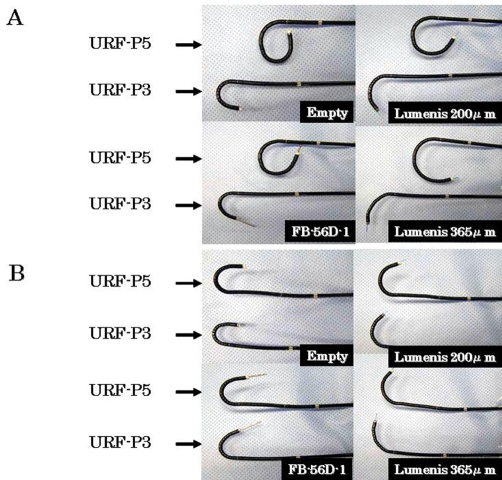
Angles of deflection of URF-P3 and URF-P5. Down angles **(A)**, Up angles **(B)**.

Renal hemangioma is a rare disease and is difficult to diagnose. Daneshmand et al. reported the effectiveness of ureteroscopy and laser treatment [1]. They used a HoYAG laser or a neodymium YAG laser in 15 cases, and 11 of them were became symptom free (mean follow periods: 20.2 months) [1]. Though recent reports demonstrate how this entity can be handled endoscopically, a significant number of these patients will require repeat surgery. Recently, our institute started to use the new and smaller-diameter, URF type P5 ureteroscope, because of its improved maneuverability. As shown in Table 1 and Figure 2, the URF type P5® is superior in its angle of deflection compared to the URF type P3®. Both ureteroscopes are 8.9 Fr in outer diameter, but the tip diameter of the URF type P5® is 5.3 Fr, smaller than that of the URF type P3®, and therefore, smooth insertion can be expected. Kourambas et al. reported that 58% of cases needed dilation of the urinary tract [2]. The present case, however, did not require dilation of the urinary tract using a ureteral access sheath.

**Table 1.** Angles of deflection of URF-P3 and URF-P5

	URF-P3	URF-P5
Device	Down/Up (°)	Down/Up (°)
Empty	185/175	275/180
Niic^a)^ 250 μm	155/160	265/165
Niic 300 μm	135/135	240/145
Lumenis^b)^ 200 μm	155/145	245/150
Lumenis 365 μm	100/100	200/110
FB-56D-1^c)^	155/150	260/165

* It is not guarantee value.

a) Niic: Laser probe (Niic, Japan).

b) Lumenis: Laser probe (Lumenis, USA).

c) FB-56D-1: 3Fr biopsy forceps (Olympus^TM^, Japan).

The URF type P5® has a smaller diameter than the URF type P3®, but has the same irrigant flow rate of approximately 37 ml/min. However, as others have reported [3], with an increase in the number of uses, the decrease in irrigant flow rate and angle of deflection have to be taken into account. In addition, the smaller the diameter, the more faults that can occur, and an increase in cost may become a cause of concern [4]. This needs to be kept in mind when treating patients.

## Conclusion

We report on the use of the new-generation flexible ureteroscope, URF type P5® (Olympus^TM^) in the management of a patient with a lower pole renal calyx hemangioma which was previously inaccessible.

## List of abbreviations

CT: Computed Tomography
MRI: Magentic Resonance Imaging
